# Cod-Liver Oil

**Published:** 1847-09

**Authors:** 


*Cod-Liver Oil.*—M. Bretonneau has come to the conclusion, after many trials, that common train oil is quite as efficacious in struma and other cases in which it is given, as the cod-liver oil. This is an important fact, if true, and demonstrates that the virtues of the latter oil are to be ascribed to its fatty principles, not to the small portion of iodine contained in it.—*Ibid, from Bulletin de Thérapeutique.*

